# What Currency Do Bumble Bees Maximize?

**DOI:** 10.1371/journal.pone.0012186

**Published:** 2010-08-16

**Authors:** Nicholas L. Charlton, Alasdair I. Houston

**Affiliations:** School of Biological Sciences, University of Bristol, Bristol, United Kingdom; Trinity College Dublin, Ireland

## Abstract

In modelling bumble bee foraging, net rate of energetic intake has been suggested as the appropriate currency. The foraging behaviour of honey bees is better predicted by using efficiency, the ratio of energetic gain to expenditure, as the currency. We re-analyse several studies of bumble bee foraging and show that efficiency is as good a currency as net rate in terms of predicting behaviour. We suggest that future studies of the foraging of bumble bees should be designed to distinguish between net rate and efficiency maximizing behaviour in an attempt to discover which is the more appropriate currency.

## Introduction

Because they are efficient wild pollinators, bumble bees are of economic and ecological importance [1]. Part of what makes them effective pollinators is their foraging behaviour. It has been argued that these animals are highly suitable for investigating foraging [2], [3], [4] and have been used so for many years [2], [4], [5], [6], [7]. They are easy to observe while foraging and appear to follow rules when visiting floral resources [2], [4], [6], the time spent flying and handling flowers can be measured [2], [6], [8], metabolic rate during activity has been calculated using experiments in the lab [9], and energy intake in the form of nectar can be measured and manipulated [6].

To study bumble bee optimal foraging, mathematical models are often used to make predictions which can then be tested [10]. A fundamental step in building a model of optimal foraging is deciding on an appropriate currency. The biological justification of a currency is that maximizing it maximizes fitness. Net rate of energetic intake has been used as the currency in many models of foraging (see [10] for a review). Alternative currencies have included gross rate and efficiency [11], [12], [13], [14], [15], [16], termed energetic quotient in [17].

Studies of bumble bee behaviour have given support to the view that they maximize net rate of energetic gain while foraging [5], [6], [8]. Subsequent work on honey bees [13], [18] showed that their behaviour was predicted more accurately by maximizing efficiency than by maximizing net rate. In this paper we examine four papers on the foraging of bumble bees [2], [5], [6], [8] to see if the observed behaviours are consistent with the maximization of efficiency.

## Methods

The models used to predict bumble bee foraging behaviour from four papers [2], [5], [6], [8] were re-arranged to allow predictions to be made based on efficiency as a currency rather than net rate of energetic intake. The results of the original and new models were compared.

### Net rate of energetic intake

Net rate of energetic intake *N* is energetic gain *E* minus energetic cost *C* divided by time *T*:
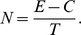
(1)


It has been argued that maximizing net rate will maximize fitness in bumble bees because it is likely that the success of the colony is strongly dependent on the amount of nectar collected by workers [19], [20] and that they are constrained by the time in which to collect resources to help produce young [2]. Additionally to nectar, floral temperature can act as a reward to bumble bees foraging [21] and Rands and Whitney [7] have shown that this can influence the behaviour that maximizes net rate.

### Efficiency

Efficiency *Q* is the ratio of energetic gain to energetic cost:
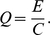
(2)


This is equivalent to the efficiency currency used by Schmid-Hempel *et al.* [13]. Maximizing efficiency gives better predictions than maximizing net rate in some situations (in honey bees: [13], [18]; in birds: [22]; see also [23]).

## Results

### Example 1

Best and Bierzychudek [8] produced a model predicting the optimum number of flowers to visit on a vertically arranged inflorescence (*Digitalis purpurea*) with decreasing nectar reward moving up the flowers on the stem. In their model (Eq. 3), it was assumed that the bees were aiming to maximize their net rate of energetic intake *N*

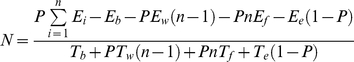
(3)


*P* = probability of encountering full flowers,

*E_i_* = expected energetic gain for the *i*th flower position,

*E_b_* and *T_b_* = energy and time costs of flying between two plants,

*E_w_* and *T_w_* = energy and time costs of flying between two flowers on the same plant,

*E_f_* and *T_f_* = costs of emptying full flowers,

*E_e_* and *T_e_* = costs of handling empty flowers,

*n* = last flower position visited.

By separating the elements of equation 3, energetic gain *E*, energetic cost *C* and total time *T* can be written as
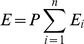
(4)

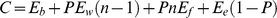
(5)

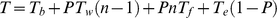
(6)Equations 4 and 5 can be arranged to give the equation for calculating efficiency *Q*

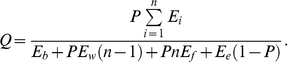
(7)


**Figure 1:**
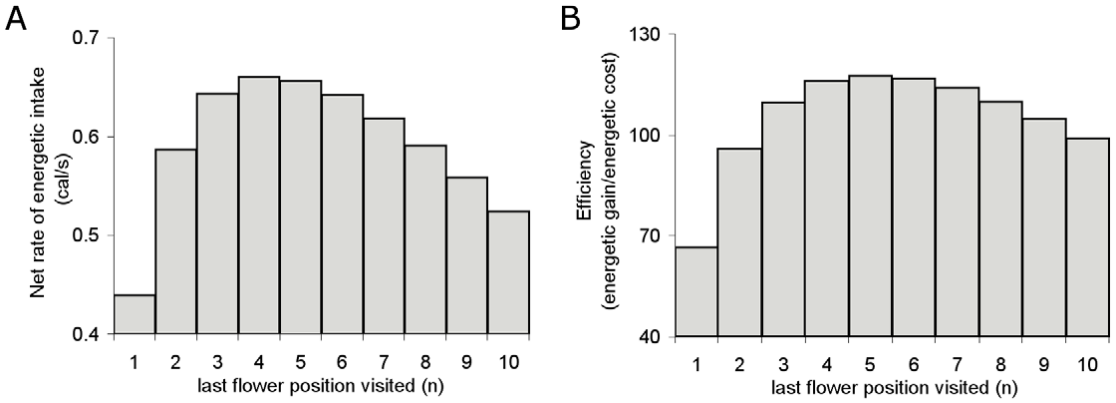
Last flower position visited for two different currencies. (a) Adapted from Best and Bierzychudek [13], showing the net rate of energetic intake when a bumble bee leaves an inflorescence at different flower positions; (b) Model based on Best and Bierzychudek [13] showing the efficiency when a bumble bee leaves an inflorescence at different flower positions.

By plotting the net rate of energetic gain *N* against flower position, it was predicted that it is optimal to leave the inflorescence after visiting flower position 4 (Fig. 1a) because this maximizes *N* [8]. Best and Bierzychudek [8] varied parameter values by 1.75 or 2 standard deviations, which produced a change in predicted flower position from 4 to 5. Their observations of the behaviour of bumble bees (*Bombus flavifrons dimidiatus*) foraging on *Digitalis purpurea* supported this prediction with the bees leaving after visiting a mean flower position of 4.55.

When the model used by Best and Bierzychudek [8] was re-arranged to calculate efficiency rather than net rate (Eq. 7), it produced a similar result with the optimal flower position being 5 (Fig. 1b). Varying *P* between 0.25 and 0.5, as is done by Best and Bierzychudek, gave optimal flower positions of 6 and 4 respectively. Altering time flying between plants or time emptying flowers has no effect on the efficiency model.

### Example 2

Hodges [6] used a model to predict when bumble bees (*Bombus appositus*) should leave a multiflowered plant (*Delphinium nelsoni*) depending on the nectar volume found in the first flower visited on that plant. The model involved calculating the net rate of energetic intake from nectar minus the energetic costs of flight and probing, divided by the total time of flying to and probing the flower (Eq. 8).
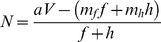
(8)


*a* = number of calories per µl of nectar

*V* = volume of nectar in a flower

*m_f_* = caloric cost per second of flight

*f* = flight time to flower

*m_h_* = caloric cost per second of probing

*h* = probe time on a flower

If we use the terms from equation 1, the elements of equation 8 can be written as
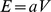
(9)

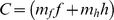
(10)

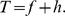
(11)From equations 9 and 10 and the definition of efficiency
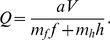
(12)


Hodges estimated a net rate of gain of 0.35 calories per second for bumble bees foraging on unmanipulated plants. This value was then used to calculate the critical volume of nectar in the first flower visited *z*, below which the bee is expected to leave the plant. The critical volume of nectar was 0.25 µl. Using the same values in the efficiency model as used by Hodges to calculate net rate produces a critical nectar volume of 0.19 µl.

Hodges predicted that when bees forage from a flower with less than 0.25 µl of nectar, they should leave that plant, but should visit the next flower on the same plant if the volume of nectar is above 0.25 µl. He tested this prediction with experimental plots of manipulated plants with bottom-most flowers containing 0, 0.5 or 1.5 µl of nectar. When Hodges looked at the frequencies of bees staying and leaving plants compared to the volume of nectar in the first flower visited he found a significantly greater number of bees staying on the plant when the volume of nectar was 0.5 µl or greater than when the volume was 0 µl, therefore supporting his prediction. If efficiency is used as the currency (*z* = 0.19 µl) instead of net rate (*z* = 0.25 µl), the prediction made by Hodges would still be supported.

### Example 3

Pyke [5] investigated foraging by *Bombus flavifrons* workers on a patch of *Aconitum columbianum* and a patch of *Delphinium barbeyi* and calculated the net rate of energetic intake at each patch. The model was slightly different to the previous examples because Pyke calculated gross rate of energetic intake *G* and rate of energy expenditure *X* then subtracted *X* from *G* to give the net rate *N*.

To calculate efficiency, gross rate of gain *G* was divided by rate of expenditure *X*:
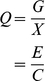
(13)


Pyke predicted that if bees are foraging from two patches of different flowers and they are maximizing their net rate of energetic intake, then the rates of intake in the two patches should be equal. If the net rate was greater in one patch than the other, the bees would move to the patch with the higher rate until both patches were equal, cf. ideal free distributions [24].

Pyke measured the gross rate of energy gained and rate of energy expenditure in a patch of *Aconitum* and in a neighbouring patch of *Delphinium* in 1977, and again in 1978. The net rates of gain between the two patches were not significantly different from each other in each year, therefore supporting Pyke's prediction.

If it is assumed that bees are maximizing efficiency, the same prediction can be made. If the ratio of energy gained to energy costs is higher in one patch, bees would switch patches until ratios in both were equal, thereby equalising efficiency in both patches.

Efficiency was calculated for each patch in each year using the values given by Pyke [5]. In 1977 the difference in efficiency values between patches was very low at 0.04 and was very similar to the difference in net rate values of 0.02. In 1978 the difference in efficiency values between patches was larger at 0.48, compared to 0.17 for net rate. The variance in 1978 was much larger than in 1977 which may have contributed to this greater difference. It was not possible to calculate variance values from the data given by Pyke [5] to test whether the differences in 1978 were significant or not.

### Example 4

Pyke [2] studied *Bombus appositus* workers foraging for nectar from *Aconitum columbianum*. He compared the net rate of energetic intake for several rules of movement and predicted that bees will employ the rule which maximizes the net rate. The data also included values for gross rate of gain *G* and rate of expenditure *X* for each rule (Table 1). Using these data, the efficiency *Q* for each movement rule was calculated (Table 1).

**Table 1:** Movement rules and their values using four currencies.

Movement rules	*G*	*X*	*N*	*Q*
1. Visit every flower in sequence along the inflorescence.	0.117	0.010	0.107	11.7
2. Choose the closest flower in the same direction as the initial direction.	0.117	0.010	0.107	11.7
3a. Always choose the closest flower.	0.113	0.010	0.103	11.3
3b. Always choose the closest flower not just visited.	0.116	0.010	0.106	11.6
3c. Always choose the closest flower that is not one of the last two flowers visited.	0.117	0.010	0.107	11.7
3d. Always choose the closest flower not previously visited.	0.117	0.010	0.107	11.7
4. Observed sequences of flowers visited.	0.117	0.010	0.107	11.7

Results showing gross rate *G*, rate of costs *X*, and net rate *N* values taken from Pyke [2] for different movement rules. Efficiency *Q* values were calculated from these data.

Movement rules 1 to 3 are rules described by Pyke and rule 4 is the observed rule that bees were following. Pyke explained that rules 3a and 3b (Table 1) have lower net rates because they include costly visits to flowers already visited, and therefore the currency is not maximized by these rules. Pyke explains that rule 3d is closest to the observed movement rule. The gross rate, net rate and efficiency values for rules 1, 2, 3c and 3d are all indistinguishable from the equivalent currency from the observed movement rule (Table 1). From these data, it appears that the currency chosen does not influence the conclusions made and the same predictions would be supported using gross rate, net rate or efficiency.

## Discussion

The foraging behaviour of honey bees is in agreement with the maximization of efficiency rather than net rate [13], [18]. Earlier work on bumble bees [2], [5], [6], [8] suggested that they maximized net rate. We have re-analysed these studies and have found that efficiency as the currency is as good a predictor as net rate of energetic intake. We have not highlighted any problems with the examples used, but have taken the results at face value.

It is not clear why honey bees should maximize efficiency. A forager that maximizes efficiency has a lower rate of gain and a lower rate of energy expenditure than a forager that maximizes net rate [14]. This may be important if energetic expenditure is costly (cf. [13], [25], [26]). Another suggestion is that bees have a fixed limit to their lifetime flight time or energy expenditure [27], and maximizing efficiency would use this constraint most effectively [28]. This maximizes the energy brought to the hive, but does not necessarily maximize the net rate at which workers are produced [29]. Two studies on different ant species did not investigate predictions based on net rate and efficiency but did consider the importance of time and energy. In the case of *Pogonomyrmex* species, energetic costs of foraging are very low [30], whereas in *Formica rufa*, it has been argued that energy costs are more important than time costs [31].

Although both honey bees and bumble bees share a number of features which may affect foraging strategies, including exploitation of the same resources, central place foraging, and provisioning the colony as well as themselves, there are a number of general differences between honey bees and bumble bees which might provide reasons for a different currency being maximized. The size of workers can vary considerably in bumble bee colonies [1], [32], whereas honey bee workers vary little [33], and worker size has been shown to affect foraging success in the form of nectar foraging rate [34]. Honey bees show a clear age or temporal polyethism, with young adult workers providing care within the nest and older workers foraging, whereas bumble bee workers do not [35]. This may result in different foraging strategies if longevity influences foraging behaviour [36]. Honey bees generally have larger colonies than bumble bees and colony size can have an affect on foraging behaviour [29].

While it has been claimed that maximizing efficiency will maximize net energetic gain if there is a limit to the energy assimilated [37], other studies suggest that a modified form of efficiency, which includes the rate of expenditure during the time when foraging is not possible, maximizes net energetic gain [38], [39].

The issue of which currency is being maximized may be further complicated by colony effects. Changes to foraging behaviour may be caused by changes to the condition of the colony [40] or colony size [29]. These changes in strategy may result in the observed behaviour being better predicted by a different currency. Similarly, the appropriate currency may change depending on the time of year [41].

The scale of measurement used to test the predictions of foraging models can be of particular importance. Hodges [6] used a rough scale of 0, 0.5, and 1.5 µl of nectar in experimental plots in trying to test the prediction that bumble bees should leave flowers with less than 0.25 µl. With efficiency used as the currency the critical volume was 0.19 µl, a relatively small difference, and one that would not be distinguished by the scale used by Hodges. Similarly, Best and Bierzychudek [8] produced a model based on the numbers of flowers visited on vertically arranged inflorescences. Bumble bees were limited by the number of flowers they could visit and all bees had to leave after visiting between 1 and 10 flowers. Again this scale may not be fine enough to distinguish conclusively between net rate and efficiency models. The tests of honey bee foraging used by Schmid-Hempel *et al.* [13] used much finer scales which made it easier to show a difference between net rate and efficiency models. The authors of early bumble bee foraging papers were not aiming to discern whether net rate or efficiency is the currency being maximized; therefore the scales they used were not inappropriate. This does, however, highlight the importance of using fine scales of measurement in future studies of bumble bee foraging behaviour.

The four papers referred to have all been cited in the classic foraging theory text by Stephens and Krebs [10] as examples in which net rate is maximized. Our analysis shows that the results are also consistent with maximizing efficiency. This highlights the importance of considering more than one currency. Further work on bumble bee foraging behaviour should focus on circumstances in which the predictions of net rate and efficiency are different.

There are two ways to proceed: quantitative predictions or qualitative predictions. Quantitative predictions are appealing, but they require accurate knowledge of key parameters such as metabolic rates [13], [18]. Houston [23] suggests that it may be more productive to make robust qualitative predictions. In general, net rate and efficiency can be distinguished by changing the energetic content of prey items. The predictions based on net rate will change whereas those of efficiency will remain the same. Future experiments could exploit this fact.
